# Supplementary material for: Association between Endocrine Therapy and Weight Gain after Breast Cancer Diagnosis among Japanese Patients: A Retrospective Cohort Study
Source: Med Sci (Basel). 2021 Jul 12;9(3):50. doi: 10.3390/medsci9030050 (PMC8293471; doi:10.3390/medsci9030050)
Supplement: Supplementary file 1 [file medsci-09-00050-s001.zip › medsci-1256220-supplementary.pdf]

Table S1. Measurement timing of each outcomes

	At diagnosis	Current
**Body mass (Primary outcome)**	○	○
Physical activity		○
Dietary intake		○
Others (income, smoking habit, etc.)		○
